# The Treatment of Neuralgia

**Published:** 1900-08

**Authors:** Robert C. Kenner

**Affiliations:** Louisville, Ky


### THE TREATMENT OF NEURALGIA.

By ROBERT C. KENNER, M.D.,

LOUISVILLE, KY.

That great neurologist Romberg declared that pain was the cry of a nerve for better blood. This statement in an axiomatic way gives us the key to causation of neuralgia. Neuralgia, in order that we may treat it with success, must be clearly understood. That is, we must find out the cause and direct our therapeutic weapons to the destruction or the removal of the cause which brought the neuralgia into being. As Romberg says, pain is the cry of the nerve for better blood. Now to treat neuralgia we must in all cases seek the cause or causes which have lowered the quality of the blood. Any disease which saps the patient's strength will put the patient in a condition to make the development of neuralgia a possibility. The causes of neuralgia may then be put down as anemia, syphilis, lithemia and the exhausting effects of all acute or chronic diseases. Again, the cause of neuralgia may be found to be the pressure of a tumor on the trunk of a nerve. Exostosis of the bones often interferes with the blood supply of the nerve and in that way produces neuralgia. Pain, neuralgia of the face, often results from exostoses of the bones of the face. These comprise in a general way the constitutional causes of neuralgia. There must be said something of the exciting causes of this affection. Exposure to cold, heat, excessive study, great excitement, grief or joy, as well as other factors, tend to produce, or rather to call into activity, an attack of neuralgia.

The treatment of neuralgia is best considered under two heads: *First.* Those measures which are directed to the removal of the constitutional factors of the causation. The *second* indication for treatment comprises measures which will relieve the patient's pain and in that secure rest.

In fulfilling the *first* indication, we shall find it important to question the patient thoroughly for information that will lead us to single out the cause which led to the development of the neuralgia. This, as said above, may be anemia, syphilis, rheumatism, lithemia, or some disease which has lowered the general health. When we have found the cause we should at once institute proper treatment. For instance, if our patient is anemic, he should be put at once on an eligible preparation of iron. If syphilis be the cause, appropriate antisyphilic treatment must be begun and continued for a time long enough to produce good results. In a great many cases of neuralgia we will find that malaria is the principal causative agent. In these cases quinine must be given regularly. In instances of malarial neuralgia we will find that there are regular recurrences of pain—these coming on with the regularity of an intermittent paroxysm. In these instances we should direct the quinine against the coming of the neuralgic attacks as we do an intermittent fever paroxysm.

Let us now consider the best means at the disposition of the profession to relieve the pain. Manifestly it is highly important to relieve the patient's suffering as quickly as that is possible. Opium is a remedy which readily suggests itself in all cases of pain. But it is well known that opium produces constipation, indigestion, loss of appetite and a great many other disagreeable results. The danger of setting up the opium habit too is never to be forgotten. The coal-tar derivatives have also been employed to relieve neuralgic pain, but the fact that these agents, as Prof. Thompson says, "are one and all muscle paralyzers," is sufficient to make the prudent practitioner exhibit these agents with wise conservatism. In all cases where there is a weak heart, or where the patient is below par physically, we should never give any of the coal-tar derivatives. We have been able to carry our point of relieving pain in these attacks of neuralgia by giving regular doses of Daniel's Conc. Tinct. Passiflora Incarnata. In doses of a teaspoonful every one, two or three hours this remedy relieves the pain and gives the patient rest. It is not a constipating remedy like the opiates; on the other hand, however, it is a mild laxative. As an anodyne, soporific and nerve stimulant there is no remedy at the disposal of the professional more prompt or more trustworthy than Daniel's Conc. Tinct. Passiflora Incarnata. It is this remedy which we now put our trust in—leaving the sedative drugs like opium and the bromides entirely out of our therapeutic armamentarium.

As yet we have said nothing of the value of heat applied over the area of the painful nerves. Hot clothes, hot salt bags, and bladders of hot water will very often greatly assist us in bringing about relief of pain.

